# Advanced Noise Indicator Mapping Relying on a City Microphone Network

**DOI:** 10.3390/s23135865

**Published:** 2023-06-24

**Authors:** Timothy Van Renterghem, Valentin Le Bescond, Luc Dekoninck, Dick Botteldooren

**Affiliations:** 1 WAVES Research Group, Department of Information Technology, Ghent University, Technologiepark 126, B 9052 Gent-Zwijnaarde, Belgium; 2 Joint Research Unit in Environmental Acoustics (UMRAE), Centre for Studies on Risks, Mobility, Land Planning and the Environment (CEREMA) and University Gustave Eiffel, F-44344 Bouguenais, France

**Keywords:** noise monitoring networks, microphones, road traffic noise, environmental noise mapping, noise indicators

## Abstract

In this work, a methodology is presented for city-wide road traffic noise indicator mapping. The need for direct access to traffic data is bypassed by relying on street categorization and a city microphone network. The starting point for the deterministic modeling is a previously developed but simplified dynamic traffic model, the latter necessary to predict statistical and dynamic noise indicators and to estimate the number of noise events. The sound propagation module combines aspects of the CNOSSOS and QSIDE models. In the next step, a machine learning technique—an artificial neural network in this work—is used to weigh the outcomes of the deterministic predictions of various traffic parameter scenarios (linked to street categories) to approach the measured indicators from the microphone network. Application to the city of Barcelona showed that the differences between predictions and measurements typically lie within 2–3 dB, which should be positioned relative to the 3 dB variation in street-side measurements when microphone positioning relative to the façade is not fixed. The number of events is predicted with 30% accuracy. Indicators can be predicted as averages over day, evening and night periods, but also at an hourly scale; shorter time periods do not seem to negatively affect modeling accuracy. The current methodology opens the way to include a broad set of noise indicators in city-wide environmental noise impact assessment.

## 1. Introduction

Road traffic is commonly the main source of exposure to environmental noise in European cities [1]. A basic step in road traffic noise mapping is gaining access to traffic parameters such as traffic intensity, vehicle speed, acceleration and traffic composition on each road segment in the network [2]. However, most traffic models focus on major roads only to perform congestion analysis during rush hours [3]. On smaller roads, in contrast, traffic data are most often lacking. Although this might be in line with the Environmental Noise Directive [4] in Europe, stipulating that noise maps should only be produced from 55 dB(A) L_den_ on, this is nevertheless problematic in view of city-wide noise mapping. When assessing human sleep disturbance due to noise, exposure mapping becomes even more challenging and should go down to levels as low as 40 dB(A) L_night_.

Knowledge of less exposed zones is relevant as well since these zones should be of primary interest for future residential developments and are potentially restorative places in a city. Furthermore, only mapping exposure in part of a city could introduce bias in environmental justice studies, an important concern nowadays when making sustainable cities (see, e.g., [5]).

An interesting line of research showed that street categorization in a city is able to estimate street-side exposure levels reasonably well [6,7,8,9,10,11], possibly accompanied with limited sets of snapshot measurements, where efforts can be minimized by suited sampling strategies [6,12]. Similarly, roadside noise measurements were shown to be able to adequately predict the underlying road traffic parameters such as vehicle speed, traffic intensity and the share of heavy vehicles [13]. In the open GIS initiative Open Street Map (OSM), every road in a city is present and assigned a specific category. This opens possibilities for full city noise mapping, including low(er) exposure zones.

Linking noise exposure maps to human health effects is currently not very successful. For an important noise policy indicator such as self-reported noise annoyance, less than 30% of the observed variance found in a surveyed population is actually captured [14]. A possible reason for this low predictive power is that current noise mapping initiatives focus on long-term equivalent sound pressure levels only. This undermines a noise map as an efficient urban sound planning instrument. Only recently, the shortcomings of the commonly used energetically equivalent levels have been officially acknowledged [15].

The way people perceive environmental noise is much more complex than what can be quantified with these standard energetically averaged sound pressure levels. A wider set of noise indicators and psycho-acoustical indicators have long been used in other contexts, e.g., in product design [16] and soundscape studies [17,18]. Essentially, people are very good listeners, and even subtle changes in the spectro-temporal content of a sound might impact the perception and reaction to it. Noise indicators of potential interest are statistical sound pressure levels, the number of events and indicators describing the dynamic nature of urban sound. Currently, city-wide mapping of such noise indicators is very scarce. A few measurement-based initiatives can be found, where walkers equipped with microphones scan a particular city quarter [19,20,21]. The use of these more advanced indicators to better predict the impacts of environmental noise is currently underexplored.

In this work, a methodology is described to predict both equivalent sound pressure levels and a wide range of other noise indicators, by means of deterministic noise modeling, where the accuracy of the predictions is improved by fitting to long-term measurements of a city noise monitoring network in a final step. The state-of-the-art deterministic noise modeling procedure, facilitating the calculation of dynamic noise indicators, is described in brief in Section 2. The proposed methodology is illustrated for the city of Barcelona (Spain) in Section 3, where a city-wide microphone network has been operational for more than a decade.

## 2. Deterministic Noise Mapping Procedure

### 2.1. Linking Traffic Data and Open Street Map Road Categorization

Street categorization data were directly used from Open Street Map (OSM). Each street category was assigned a set of plausible traffic parameters (more precisely traffic intensity, vehicle speed and share of heavy vehicles). This assignment starts from existing (highway) traffic count databases, and it is ensured that the expected logics such as a lower traffic intensity, lower vehicle speed and a lower share of heavy vehicles on minor streets compared to major streets are present. Depending on the deterministically predicted noise indicators corresponding to a given scenario, additional scenarios were manually added. In total, 15 scenarios were used (see Appendix A for an overview of the parameter settings). In a final step, the calculated outcomes for a wide set of noise indicators are weighted to minimize the difference with measurement from the microphone network, as will be discussed in Section 3.2.

### 2.2. Dynamic Traffic Model

Simplified vehicle movements are modeled based on the hourly averaged number of vehicles and their speeds. Vehicles are launched on a road segment at a fixed speed. When reaching the end of that segment, the vehicle is removed from the simulation, meaning there is no vehicle transfer from one segment to another. The inter-vehicle times respect a Poisson distribution, and vehicle speeds of the different cars follow a normal distribution. At the end of the simulated hour, at each road segment, the (static) vehicle counts and average speeds are respected. More information on this simplified micro-simulation traffic procedure can be found in [22]. A time step of 1 s was considered in this work.

### 2.3. Traffic Noise Emission Model

Vehicle category, number of vehicles per hour, and average speed are used as input for the CNOSSOS [23] road traffic acoustic emission model. These traffic-related inputs come from the dynamic traffic modeling procedure described in Section 2.2.

### 2.4. Sound Propagation Model

The sound propagation modeling procedure combines the CNOSSOS sound propagation model [23] with aspects from the QSIDE urban sound propagation model [24]. Vehicles close to a receiver (within a radius of 500 m) are treated differently from those further away (between 500 m and at maximum 2000 m).

At close distance, and when a direct line-of-sight propagation path is possible between a source and a receiver, geometrical divergence, ground effect and atmospheric absorption are included following the CNOSSOS sound propagation model, implemented in the open access NoiseModelling framework [25,26]. Only in the absence of a line-of-sight propagation path, diffractions around horizontal edges and reflections on vertical objects are accounted for. The maximum reflection order is 2, and the maximum source-reflection distance is 50 m (which are standard settings; see, e.g., [27]). A standard noise mapping receiver height of 4 m is used.

Scattering on atmospheric turbulence is added to the attenuation factors to avoid levels becoming unrealistically low, especially behind objects. The QSIDE engineering scattering approach [28] was used, providing an easy-to-evaluate expression adapted to the urban environment, accounting for sound frequency, propagation distance, street canyon geometry and turbulence strength. Although the model could include local street canyon geometry in detail, standard building heights and widths were used to avoid time-demanding retrieval from geographical input data. Turbulence structure parameters C_v_^2^ and C_T_^2^ (see Table 1) depend on whether the propagation occurs in rural/suburban settings or in the dense urban fabric, and during the daytime or at night. These turbulence parameters are based on long-term observations over flat rural zones with dispersed smaller cities [29]; in the dense urban environment, turbulence strength is doubled in a simplified approach.

In order to capture dynamic noise indicators and noise events, considering individual nearby vehicles is essential. This is not the case anymore for road traffic further away contributing mainly to the background noise at a receiver. This allows bundling the acoustic energy of cars in a limited number of emission points as optimized by the NoiseModelling framework. For the propagation simulations, an approach similar to that for nearby traffic is followed, so depending on whether a line-of-sight path is possible or not.

The CNOSSOS favorable sound propagation approach (i.e., downward refraction) is only considered in the absence of line-of-sight paths. In the CNOSSOS simplified curved ray approach, the difference between refraction/no refraction is mainly relevant in the case of propagation over objects. The probability of favorable sound propagation is then set to 50% in any propagation direction.

## 3. The Barcelona Microphone Sensor Network

### 3.1. Measurements and Data Handling

The Barcelona microphone measurement network is unique in its kind due to its size (roughly 250 monitoring points spread over the city) and since it has been operational for more than a decade. The network contains both fixed sensors and sensors that are repositioned period-wise to maximize the zone monitored. Together with the fact that individual sensors are prone to accidental failure, the dataset is rather discontinuous in nature. Nevertheless, at some fixed sensors, continuous sound pressure level measurements over several years are present.

The sensors are opportunistically positioned, e.g., directly attached to window sills or near balconies. This means that the extent to which façade reflections impact the sound pressure level measurements is not fixed (see Section 4). Microphones are always facing the streets and are representative of the most exposed building side.

To limit the impact of changes in the traffic network infrastructure and its management (such as limiting traffic in specific streets, changing the direction of circulation, ban of heavy traffic), a 3-year period was selected, which was a compromise between keeping this period as short as possible and having a sufficient amount of data while keeping as many sensor locations as possible for processing. Nevertheless, changes in the traffic network cannot be fully avoided within this time frame, and if this was the case, the measurements were then the average between the two different traffic situations. Note that convergence must still be reached at such locations (see next paragraph) for a microphone position to be used.

The processing of the measurement sensors was performed as follows. A basic time period of 15 min was chosen for all indicators. Previous research [12] showed that this is a suitable time frame in road traffic noise-dominated urban environments. Shorter periods could lead to difficulties in stabilizing the noise indicators, giving too much emphasis on momentary variations. When extending to longer periods, the temporal variations in the sonic environment might not be captured sufficiently.

For a sensor location to be considered in further analysis, at least 3 weeks of data (not necessarily continuous) should be available. Weekends were excluded to avoid uncommon traffic situations. As a simplified convergence criterion, the difference between taking 80% of the data and all available data (in a chronological way) should be less than 1 dB when (linearly) averaging a noise indicator that uses a decibel scale. For event-based indicators, this criterion is set to five events, and for the intermittency ratio set to 5% (see further). If this condition is not met, this sensor location is disregarded at least for a specific time period. Removing sensor data during the day period, e.g., does not necessarily mean that the sensor location is also disregarded during the evening and night periods.

The measurement network contains sensors with two levels of detail. Most microphone stations report total A-weighted sound pressure levels with a basic integration period of 1 min. These data were available at 93 sensors in the current study (see Section 3.3), during the period 2020–2021–2022. Secondly, measurement stations logging 1/3-octave bands with a basic integration period of 1 s were used during the period 2016–2017–2018. These more detailed data were available at 23 stations and allowed calculating more advanced noise indicators as discussed in Section 3.4.

### 3.2. Machine Learning Fitting Procedure

As an example supervised machine learning fitting algorithm, an artificial neural network was used, as implemented in Matlab [30]. A standard split into training, validation and test sets using 70%, 15% and 15% of the data, respectively, was chosen. The training algorithm “Levenberg–Marquardt backpropagation” was used, which is recommended as a fast, first-choice procedure [30]. To prevent overfitting, only five neurons were used, with a single hidden layer [31]. Given the random split into training, validation and test datasets, models were repeatedly constructed, allowing the use of averaged model predictions and giving an indication of confidence intervals on repeated predictions.

In the case of predicting A-weighted equivalent sound pressure levels (see Section 3.3), the input consists of 93 locations × 15 traffic scenarios; there are 93 (locations) × 3 (day, evening and nightly averaged) or 93 (locations) × 24 (hourly averaged) outputs. In case more advanced noise indicators were included (see Section 3.4), 23 (locations) × 15 (traffic scenario) × 29 (indicators) inputs were used to predict 23 (locations) × 29 (indicators) × 3 (day, evening and nightly averaged) outputs.

This work does not aim at finding the most accurate or fastest machine learning approach for this specific application, but rather showcases what can be achieved with a standard and well-established supervised machine learning fitting approach. Similarly, further optimization of the neural network settings is also beyond the scope of the current work.

### 3.3. Predicting A-Weighted Equivalent Sound Pressure Levels

Using the basic L_Aeq,1min_ values, an integration is performed to 15 min. In the next step, L_Aeq,15min_ data are linearly averaged over day (7:00–19:00), evening (19:00–23:00) and night (23:00–7:00) periods, thus providing a typical value in each period, and form the basis for the artificial neural network predictions. In a second set of predictions, hourly averaged L_Aeq,15min_ data are used as well.

**Figure 1:**
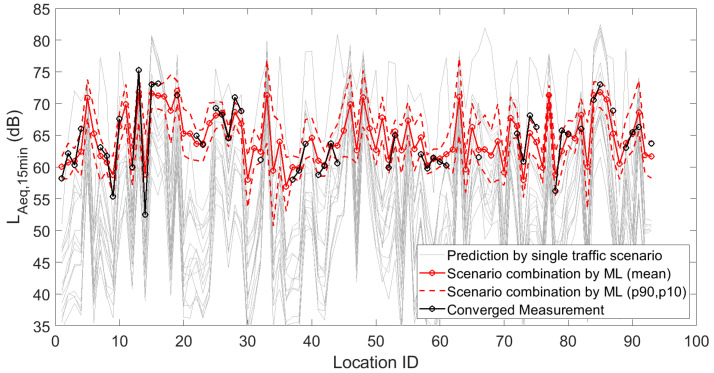
Deterministic predictions of L_Aeq,15min_ for each single traffic scenario, by the artificial neural network (showing the mean prediction and the 90th and 10th percentiles, based on repeated model constructions), together with the converged measurements, at each of the 93 locations where a sensor node is/was operational. Data shown here are the linearly averaged L_Aeq,15min_ during the daytime.

**Figure 2:**
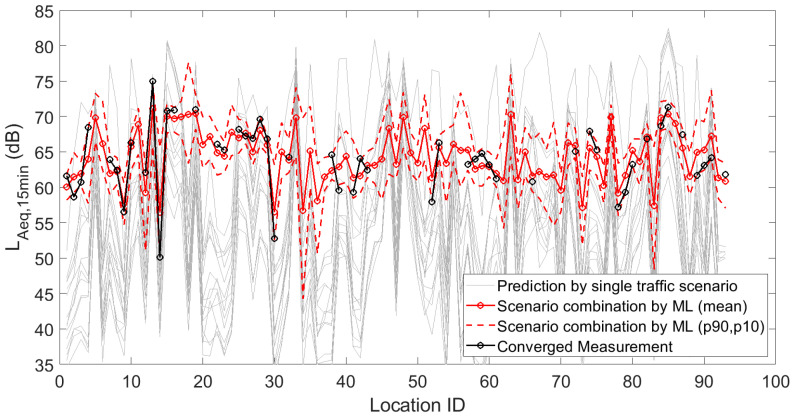
Deterministic predictions of L_Aeq,15min_ for each single traffic scenario, by the artificial neural network (showing the mean prediction and the 90th and 10th percentiles, based on repeated model constructions), together with the converged measurements, at each of the 93 locations where a sensor node is/was operational. Data shown here are the linearly averaged L_Aeq,15min_ during the evening.

**Figure 3:**
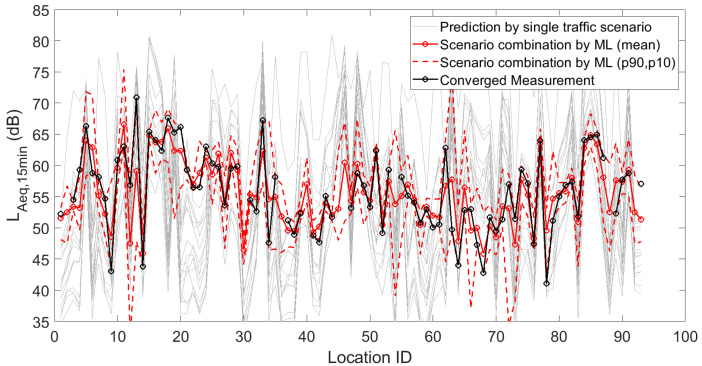
Deterministic predictions of L_Aeq,15min_ for each single traffic scenario, by the artificial neural network (showing the mean prediction and the 90th and 10th percentiles, based on repeated model constructions), together with the converged measurements, at each of the 93 locations where a sensor node is/was operational. Data shown here are the linearly averaged L_Aeq,15min_ during the night.

Figure 1, Figure 2 and Figure 3 depict the 15 deterministic predictions at each sensor location that formed the basis for the weighting by the artificial neural network, together with the measured values (i.e., the ground truth), the mean predicted values and the 90th and 10th percentiles based on repeated model constructions. On the horizontal axis, the location ID number is used, which is an arbitrary number but easily allows assessing changes from location to location, both in measurements and predictions. Figures are shown for the daily, evening and nightly averaged L_Aeq,15min_. Note that sensors with an insufficient number of data points or sensors not leading to converged indicators (see Section 3.1) were obviously not used during the construction of the machine learning model. Once the model was constructed, predictions with the model were performed at all 93 sensor locations. Clearly, only locations with both measurements and predictions were considered in the subsequent accuracy analysis.

**Figure 4:**
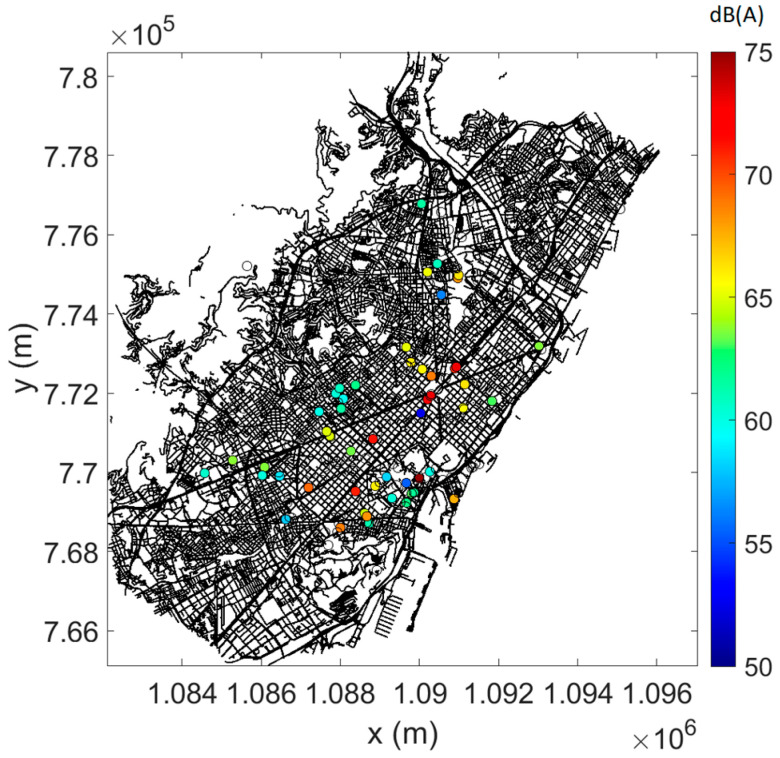
Linearly averaged L_Aeq,15min_ from measurements during daytime. Only sensor locations with converged measurements and data falling within the pre-selected timeframe are shown.

**Figure 5:**
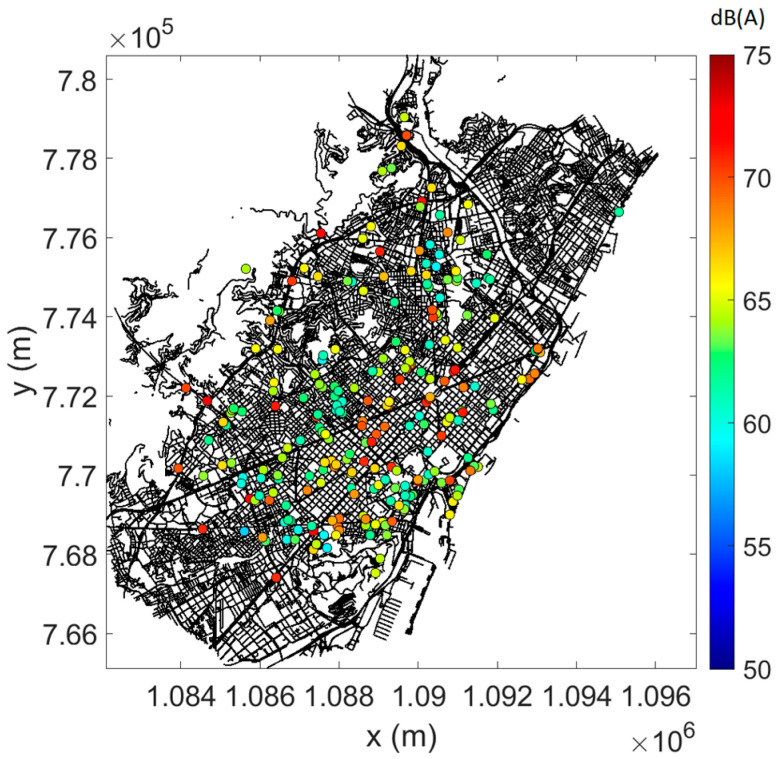
Mean L_Aeq,15min_ predictions during daytime, at 93 spots where a sensor node is/was operational.

**Figure 6:**
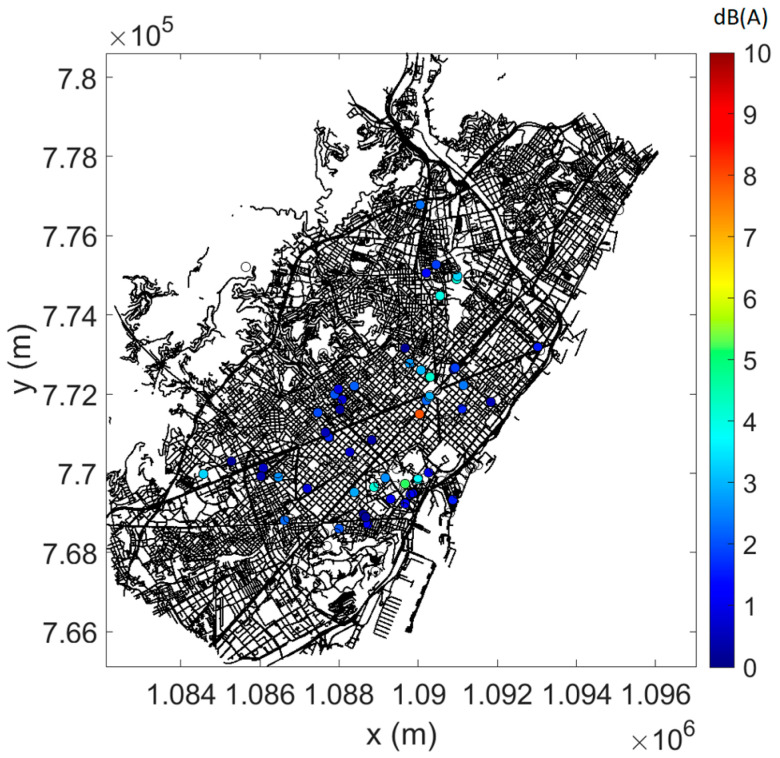
Root-mean-square error (RMSE) between measured and (mean) predicted L_Aeq,15min_ during daytime. Only sensor locations with converged measurements and data falling within the pre-selected timeframe were used for this analysis.

In Figure 4, Figure 5 and Figure 6, the measured data are plotted on the street map of Barcelona, complying with the selection criteria (see Section 3.1), the (mean) predictions at (all) sensor locations, and the difference between measurements and predictions where possible (as root-mean-square error, RMSE). As an example, daytime data only are shown. At most locations, prediction errors are limited, although a few points give rise to larger errors.

**Figure 7:**
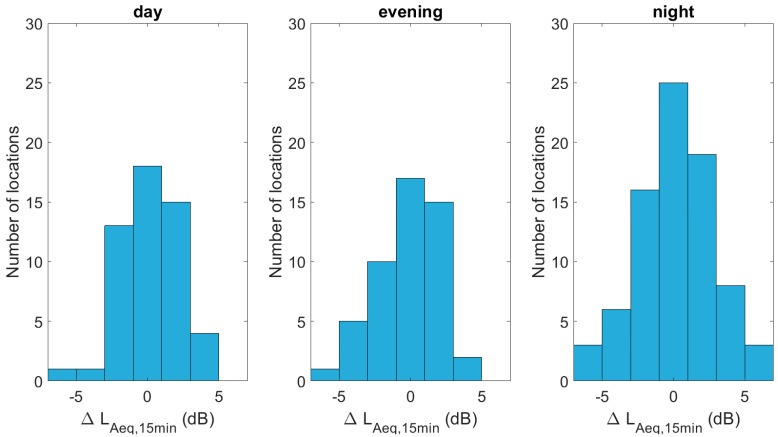
Histograms showing the difference between the (mean) predicted and measured L_Aeq,15min_, linearly averaged over daytime, evening and night hours.

The histograms in Figure 7 depict the actual differences between measurements and predictions, showing that the zero error class is most populated in all time periods considered. During the night, the distribution is still symmetrical, but the spread seems somewhat larger. The RMSEs are 2.0 dB(A) during the daytime, 2.1 dB(A) during the evening and 3.3 dB(A) during the night.

**Figure 8:**
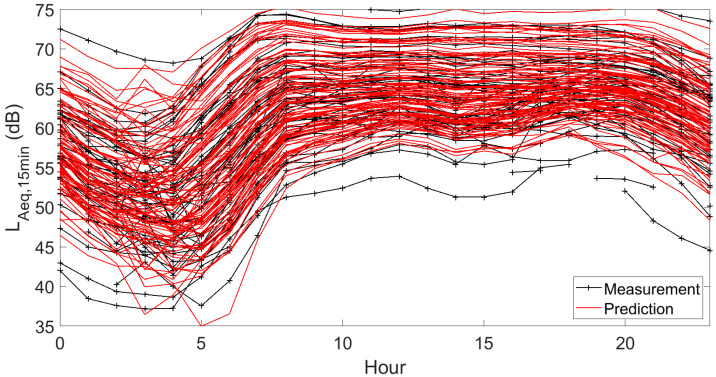
Hourly temporal patterns of L_Aeq,15min_ at all 93 measurement locations. The measurements are shown together with the mean predictions based on repeated model construction. Data shown here are the linearly averaged L_Aeq,15min_ during a specific hour. Interrupted lines indicate hours where measurements are not converged due to an insufficient amount of data.

**Figure 9:**
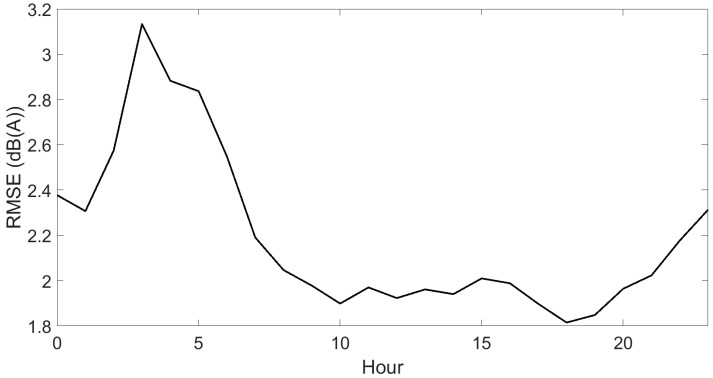
Root-mean-square error (RMSE) between measured and (mean) predicted L_Aeq,15min_ over all locations on an hourly basis. Only sensor locations with converged measurements and data falling within the pre-selected timeframe are considered.

Results for hourly predictions are depicted in Figure 8, shown as temporal patterns over 24 h periods at each sensor location. The measured temporal patterns are shown as well. This figure does not allow the comparison of measurements and predictions at any individual sensor location, but it nicely shows that the bulk of the temporal patterns are well predicted. Both locations with a rather flat pattern and those with stronger level drops during the night hours can be distinguished, both in the measurements and predictions. Directly related to Figure 8, Figure 9 shows the hourly RMSEs. Minimum values are found around noon, near 2 dB(A), and increase slightly above 3 dB(A) between 3 and 4 o’clock at night.

### 3.4. Predicting Advanced Noise Indicators

The spectro-temporal detail of the more detailed measurement stations is 1 s and in 1/3-octave bands. All indicators are first evaluated over a 15 min period. Calculating over longer time frames (hourly, or day/evening/night period) is performed by linearly averaging the 15 min indicators.

In Table 2, an overview is given of the noise indicators that were considered in this work. It concerns the basic equivalent sound pressure levels, either non-weighted (L_eq_), A-weighted (L_Aeq_) or C-weighted (L_Ceq_). For the calculation, the basic 1 s 1/3-octave bands are first frequency-weighted and integrated to a total sound pressure level, and in the next step, they are integrated to 15 min.

Statistical level L_An_ denotes the A-weighted sound pressure level exceeded n% of the time, where L_A10_, e.g., is representative of peak levels, while L_A90_, e.g., is representative of background noise levels. The percentiles are calculated over a period of 15 min based on the 1 s A-weighted total levels.

ENx is the number of events above a fixed L_Aeq_ of x dB. An event is defined by a peak in the time history of total A-weighted levels, lasting for at least 1 s. The number of occurrences is counted over a 15 min period. Similarly, the number of events above a statistical noise level, or a statistical noise level plus x dB, is considered as well.

Next, a number of sound dynamics indicators are considered. The difference between L_A10_ and L_A90_ is a commonly used metric in this respect; a large value means a strong variation in exposure level. L_C10_−L_C90_ is a similar indicator; in theory, it is applicable to higher absolute exposure levels, but it is often used to study dynamics with a focus on the low sound frequency range. These indicators are calculated as the differences between statistical levels assessed over the 15 min period.

Indicators σ_AS_ and σ_CS_ are the standard deviations on either the A-weighted or C-weighted 1 s total sound pressure levels, calculated over a period of 15 min.

The intermittency ratio IntRatio [32] is defined as the acoustic energy present in peaks relative to the total amount of energy in a particular time period, expressed as a percentage. The threshold to detect peaks is set to 3 dB (above the L_eq,15min_) as proposed in [32].

When having access to detailed spectro-temporal data, the list of noise indicators that could be calculated is clearly not limited to the current selection. The current selection could especially be relevant to acoustically characterize urban traffic noise.

**Figure 10:**
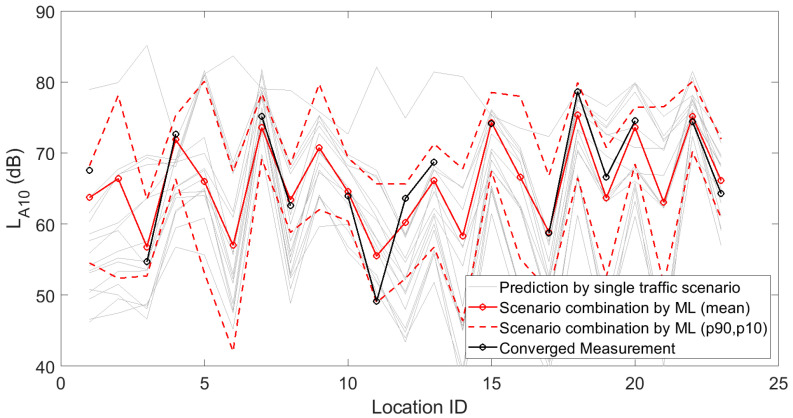
Deterministic predictions of the statistical sound pressure level L_A10_ for each single traffic scenario, by the artificial neural network (showing the mean prediction and the 90th and 10th percentiles, based on repeated model constructions), together with the converged measurements, at each of the 23 locations where a sensor node is present with detailed logging capabilities. Data shown here are linearly averaged over 15 min periods during daytime.

**Figure 11:**
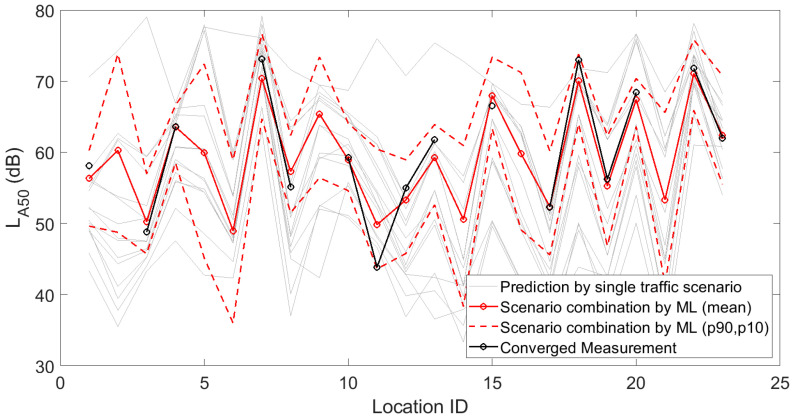
Deterministic predictions of the statistical sound pressure level L_A50_ for each single traffic scenario, by the artificial neural network (showing the mean prediction and the 90th and 10th percentiles, based on repeated model constructions), together with the converged measurements, at each of the 23 locations where a sensor node is present with detailed logging capabilities. Data shown here are linearly averaged over 15 min periods during daytime.

**Figure 12:**
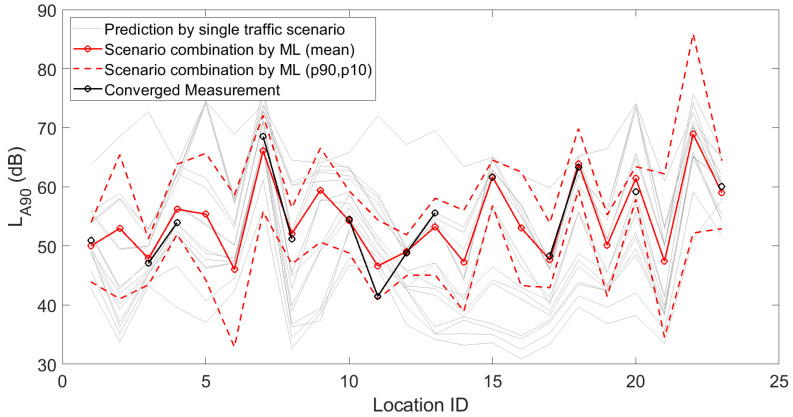
Deterministic predictions of the statistical sound pressure level L_A90_ for each single traffic scenario, by the artificial neural network (showing the mean prediction and the 90th and 10th percentiles, based on repeated model constructions), together with the converged measurements, at each of the 23 locations where a sensor node is present with detailed logging capabilities. Data shown here are linearly averaged over 15 min periods during daytime.

In Figure 10, Figure 11 and Figure 12, fifteen deterministic predictions for (a selection of) statistical sound pressure levels at each sensor location are depicted, forming the basis for the weighting by the artificial neural network. These graphs further show the measured values, the mean predicted values and the 90th and 10th percentiles based on repeated model constructions. Figures are shown for the daily averaged indicators only for brevity.

**Figure 13:**
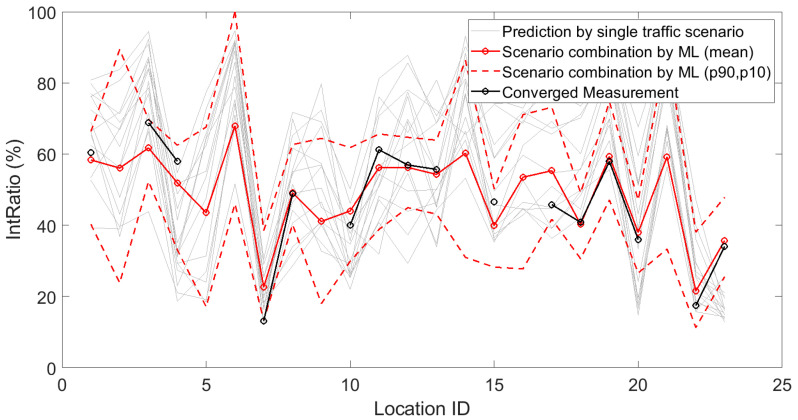
Deterministic predictions of the intermittency ratio for each single traffic scenario, by the artificial neural network (showing the mean prediction and the 90th and 10th percentiles, based on repeated model constructions), together with the converged measurements, at each of the 23 locations where a sensor node is/was operational with detailed logging. Data shown here are linearly averaged over 15 min periods during daytime.

As another example, intermittency ratio predictions can be evaluated based on Figure 13 during the daytime. An overview of the RMSEs of all 29 indicators, averaged over day, evening and night periods, is given in Table 3.

The A-weighted statistical levels, averaged over the three periods considered, only score slightly worse (2.3 dB) than the equivalent sound pressure levels (2.0 dB). The σ_S_ indicators, either A-weighted or C-weighted, have an RMSE lower than 1 dB, while L_10_–L_90_ is predicted within 2 dB.

The errors in the number of noise events shown in Table 3 should be seen relative to the total number of events at a particular location. The larger errors are typically for locations and indicators with more events. When expressing the RMSEs relative to the (measured) total number of events, values equal to 26%, 28% and 30% are found for day, evening and night periods, respectively. The IntRatio is predicted with an RMSE of about 5–6%.

## 4. Discussion

The current work shows that a basic set of deterministic noise mapping simulations, relying on street categorizations, forms a good basis for predicting sound pressure levels and related noise indicators. RMSEs with relation to 15 min equivalent sound pressure levels, averaged over day, evening and night time, were 2.0 dB(A), 2.1 dB(A) and 3.3 dB(A), respectively, for the case study of Barcelona, relying on a subset of 93 sensor locations with data over a period of 3 years. The histograms of differences between (fitted) simulations and measurements show that most data fall in the 0 dB(A) error class. Using this same approach, linearly averaged hourly predictions are possible, showing an RMSE below 2 dB(A) around noon, increasing to 3 dB(A) in the middle of the night. However, at a few sensor locations, larger errors are observed. Averaged daily temporal patterns of L_Aeq,15min_ can be predicted too, showing logical results, and clearly distinguish between sensor locations with a rather flat pattern and those with a stronger level drop during the night hours.

This same procedure also works well for the more advanced noise indicators considered here. Some care is needed since this analysis is based on a more limited number of sensors. Nevertheless, similar RMSEs were obtained, generally between 2 and 3 dB for indicators expressed in decibel units. Predicting a set of statistical sound pressure levels leads to errors similar to those for equivalent sound pressure levels. Capturing sound dynamics by means of the standard deviation, calculated based on 1 s L_Aeq_ or L_Ceq_, leads to prediction errors near 1 dB. The intermittency ratio is predicted with an RMSE of about 5–6%.

The number of events, an indicator that might be especially relevant for assessing sleep disturbance by environmental noise, can be reasonably well predicted too. The larger RMSEs are typically for locations and indicators with more events. Note that at a location with a large number of events, missing a few events will not change the perception of the sonic environment. In contrast, if there are only a few events, each individual event has a bigger importance. Median relative errors are near 30%.

The artificial neural network fitting procedure uses a random split into training, validation and test subsets. Various model constructions allow deriving prediction ranges per location as a measure for the modeling uncertainty, while averaging model responses stabilizes results. For the L_Aeq,15min_ predictions, using 93 sensor locations, the 10th and 90th percentiles on the predictions cover rather small ranges. For the more advanced noise indicator predictions (23 sensors), these uncertainty ranges are extensive and could indicate a lack of a sufficient amount of data. Notwithstanding these concerns, the linearly averaged fitted predictions make sense and do not seem to deviate more from the measurements than the artificial network trained on 93 sensor locations. Although the fitting procedure does not (explicitly) impose boundaries during the training, the average predictions nicely fit within these ranges. This indicates that the translation of street categories to traffic parameters, performed iteratively based on expert judgment, is performed adequately, at least for the specific sensor locations considered.

These observed errors should further be seen in view of façade reflections that play a significant role in inner-city street-side measurements. In the current dataset, the actual distance of the microphones relative to the façades is not fixed. Since exterior building surfaces are predominantly rigid, this will lead to strong reflections; consequently, standing waves will appear in front of a façade, characterized by frequency ranges with constructive interferences. At other frequencies, pronounced destructive interference dips appear. Measurements and numerical simulations, focusing on road traffic noise sources, showed that the increase in sound pressure level, relative to the free field, is typically between 3 and 6 dB [33,34,35,36]. Interference effects are not accounted for in the noise mapping propagation module. This difference in 3 dB could therefore be considered as a random factor during the fitting procedure. In addition, the distance relative to the driving lane is also relevant in relation to the magnitude of the façade reflections [37,38].

The deterministic modeling process is based on the CNOSSOS [23] and QSIDE models [24]. CNOSSOS is currently the recommended model for noise mapping in the European Union and basically stems from the ISO9613-2 model [39]. The model captures the basic physics of sound propagation in the outdoor environment. The focus is on rapid evaluation rather than achieving high accuracy, making the model suited for strategic noise mapping in large zones. The QSIDE model was designed specifically to model exposure at non-directly exposed building sides in urban environments. Only the QSIDE turbulent scattering formula [28] is used in the current context. The parameter settings and model choices aim at balancing the expected physical accuracy and computational cost.

In this respect, simplifications and inaccuracies arising from the deterministic modeling process might be—at least partly—corrected for by the fitting procedure, aiming primarily at weighting the level predictions of the different traffic parameter scenarios. In the current deterministic simulations, the reflection order on vertical objects is limited to 2. In a street canyon, however, a much larger number of reflections is needed; convergence in sound pressure level might need an order of 20 or more [40]. Despite this strong limitation during the deterministic modeling, street-side levels are still adequately predicted. A possible explanation is that a constant factor (“street amplification”) is implicitly added during the fitting. Research found that the many sound reflections in a street build up a reverberant field that can be captured by a “building correction” [37] or “reflection ratio” [41], mainly depending on street width [41]. Logically, street width depends on street category. As another example, the local vehicle fleet might not fully align with the standard sound emission model and might depend on the average age and maintenance degree of the cars and the popularity of specific engine types [42].

In addition, the diffraction formula used in CNOSSOS is a strong simplification of a complex physical process (see, e.g., [43]). Especially in the case of realistic urban environments, characterized by consecutive diffractions over roof edges, accuracy will further degrade. The minimum level set by accounting for turbulent scattering could at least avoid levels becoming unrealistically low, which is a common issue with simplified diffraction modeling over buildings [24]. Note, however, that concerns on modeling sound propagation towards shielded building sides might not be a main problem in the current work since most sensors are positioned at the street side. Application of the current methodology to shielded building façades, of high relevance, e.g., with respect to the promotion of quiet sides [44], needs further study. Indeed, sound fields in non-directly exposed zones in the urban environment might be strongly different, e.g., in relation to their dynamics [45].

The methodology presented in this work needs an extensive microphone measurement network for weighting the traffic scenarios and therefore is not readily applicable to any city. Although such (permanent) networks are still scarce, they are increasingly being deployed in bigger cities all over the world such as Barcelona [46], New York [47], and Paris [48], just to name a few. Although high-end network-based microphone systems are possible, more affordable options using consumer electronics microphones exist. Such sensors have become very cheap due to mass production. Although such sensors are not primarily intended as measurement devices, they can measure sound pressure levels reasonably well. It was shown before that cheap microphones that highly correlate to reference type-1 microphones even in harsh outdoor conditions can be identified; when the deviations are expressed in total A-weighted (road traffic) noise levels, values of less than 1 dB are obtained, in excess of the deviation amongst reference microphones themselves [49]. More recent developments and experience with MEMS microphones [50,51,52] could further boost the deployment of city-wide microphone networks. The implicit traffic data retrieval in the current work could further benefit from including computer vision technologies [53]. In [54], e.g., camera images were directly used for noise mapping using machine learning.

A relevant question is whether the trained fitting network could be transferable to other cities. It is expected that this is unlikely, since the link between street categories and traffic parameters could be strongly locally dependent. In addition, as discussed before, the network might not only weight the traffic scenarios, but also traffic and propagation-related aspects are corrected for. Examples are the typical street widths and number of lanes corresponding to a specific street category in a city, traffic management policy, and preferred road surfaces and their maintenance.

## 5. Conclusions

The proposed advanced noise indicator mapping procedure, using a set of deterministic predictions combined with data from a city microphone measurement network, has been shown to be an approach with high potential. Both equivalent sound pressure levels and more advanced noise indicators expressed in decibel units lead to RMSEs between 2 and 3 dB. These deviations should be positioned relative to the 3 dB variation in street-side urban road traffic noise exposure measurements when the microphone positioning relative to the façade is not fixed. The current work further shows that city-wide noise mapping without access to direct traffic data is feasible on the condition that a microphone network is available, and at the same time, systematic inaccuracies occurring at any stage during the deterministic modeling process might be implicitly corrected for, at least to some extent. Continued research and more case studies are needed to see whether the current concept can grow to a mature urban traffic noise mapping methodology.

## Figures and Tables

**Table 1:** Overview of the sound propagation models and parameter settings.

	Close-by Traffic		Far Traffic	
Radius around receiver (in m)	<500		≥500 and <2000	
Traffic (noise emission) modeling	Simplified dynamic traffic modeling following [22], at a 1 s time interval.		Aggregated traffic at discrete emission points. Number of emission points minimized by NoiseModelling [26]	
If a direct line-of-sight path is possible	CNOSSOS sound propagation model [23] without reflections on vertical objects, without diffractions, and in a non-refracting atmosphere.			
Only obstructed sound paths are present	CNOSSOS sound propagation model [23] including reflections on vertical objects (reflection order 2, maximum source-reflection distance 50 m) and including diffractions on horizontal edges. Downward refraction (“favorable conditions”) is assumed with 50% occurrence in any direction.			
	Turbulent scattering model [28]			
	Rural/suburban		Dense urban fabric	
Distance to façade (m)	Not applicable		5	
City canyon width (m)	Not applicable		15	
Building height (m)	8		20	
	Day	Night	Day	Night
C_v_^2^ (m^4/3^/s^2^)	0.4	0.2	0.8	0.4
C_T_^2^ (K^2^/m^2/3^)	0.7	0.04	1.4	0.08

**Table 2:** Overview of the 29 noise indicators considered in this work.

Equivalent Sound Pressure Levels	Statistical Sound Pressure Levels	Number of Events above x dB(A)	Number of Events above a Specific Indicator	Sound Dynamics Indicators	Intermittency Ratio
L_eq_	L_A01_	EN55	ENL_A10_	σ_AS_	IntRatio
L_Aeq_	L_A05_	EN60	ENL_A50_	σ_CS_	
L_Ceq_	L_A10_	EN65	ENL_A50_ + 3	L_A10_–L_A90_	
	L_A50_	EN70	ENL_A50_ + 10	L_C10_–L_C90_	
	L_A90_	EN75	ENL_A50_ + 15		
	L_A95_	EN80	ENL_A50_ + 20		
	L_A99_		ENL_Aeq_ + 10		
			ENL_Aeq_ + 15		

**Table 3:** Root-mean-square errors (RMSEs) for each noise indicator considered over all sensor locations, split into day, evening and night periods.

Indicator	Day	Evening	Night
L_eq_ (dB)	1.9	2.2	1.8
L_Aeq_ (dB)	2.1	2.1	1.9
L_Ceq_ (dB)	2.1	2.3	2.0
L_A01_ (dB)	2.2	2.2	2.3
L_A05_ (dB)	2.4	2.4	2.1
L_A10_ (dB)	2.6	2.5	1.9
L_A50_ (dB)	2.2	2.3	2.3
L_A90_ (dB)	1.9	2.5	2.6
L_A95_ (dB)	2.1	2.6	2.0
L_A99_ (dB)	2.1	2.7	2.0
σ_AS_ (dB)	0.9	1.0	1.1
σ_CS_ (dB)	0.8	0.8	0.9
L_A10_–L_A90_ (dB)	2.3	2.4	2.5
L_C10_–L_C90_ (dB)	1.6	1.9	1.9
EN55 (n.o.e.) ^1^	4.3	6.5	5.3
EN60 (n.o.e.)	7.4	7.0	8.4
EN65 (n.o.e.)	10.6	7.8	9.4
EN70 (n.o.e.)	12.3	10.9	2.7
EN75 (n.o.e.)	3.4	3.9	4.2
EN80 (n.o.e.)	3.2	3.1	1.6
ENL_A10_ (n.o.e.)	4.9	4.8	4.8
ENL_A50_ (n.o.e.)	8.3	9.8	8.6
ENL_A50_ + 3 (n.o.e.)	7.8	8.8	6.2
ENL_A50_ + 10 (n.o.e.)	3.9	4.2	3.9
ENL_A50_ + 15 (n.o.e.)	1.8	2.1	2.5
ENL_A50_ + 20 (n.o.e.)	0.9	0.8	1.7
ENL_Aeq_ + 10 (n.o.e.)	1.0	1.0	1.3
ENL_A10_ (n.o.e.)	0.5	0.5	0.6
IntRatio (%)	4.9	6.1	5.5

^1^ n.o.e. stands for “number of events”.

## Data Availability

This study made use of third-party data and software that are partly open. The new data created in this work is available upon request.

## Appendix A

In Table A1, the link between the Open Street Map categories and the traffic intensity, vehicle speed and share of heavy vehicles is shown, for the 15 scenarios that were used in this work.

**Table A1:** Traffic parameters assigned to the Open Street Map street categories that were explicitly calculated with the deterministic noise mapping methodology. Vehicle intensities (VIs) are expressed in cars per hour, the share of heavy vehicles (SHV) in %.

**Period**	**Road Type**	**Vehicle Speed (km/h)**	**VI (SHV)** **Scenario 1**	**VI (SHV)** **Scenario 2**	**VI (SHV)** **Scenario 3**	**VI (SHV)** **Scenario 4**	**VI (SHV)** **Scenario 5**	**VI (SHV)** **Scenario 6**
Day	motorway	130	20,400 (15%)	10,200 (15%)	5100 (15%)	20,400 (15%)	20,400 (20%)	20,400 (15%)
	trunk	110	8400 (15%)	4200 (15%)	2100 (15%)	8400 (15%)	33,600 (20%)	16,800 (15%)
	primary	80	4800 (0%)	2400 (0%)	1200 (0%)	4800 (0%)	19,200 (5%)	9600 (0%)
	secondary	80	3300 (0%)	3300 (0%)	3300 (0%)	1750 (0%)	26,400 (5%)	6600 (0%)
	tertiary	50	350 (0%)	350 (0%)	350 (0%)	175 (0%)	8400 (0%)	2100 (0%)
	residential	30	175 (0%)	175 (0%)	175 (0%)	85 (0%)	350 (0%)	1400 (0%)
	service	30	80 (0%)	80 (0%)	80 (0%)	42 (0%)	175 (0%)	175 (0%)
Evening	motorway	130	20,400 (11%)	10,200 (11%)	5100 (11%)	20,400 (11%)	20,400 (16%)	20,400 (11%)
	trunk	110	1600 (11%)	800 (11%)	400 (11%)	1600 (11%)	12,800 (16%)	3200 (11%)
	primary	80	1000 (0%)	500 (0%)	250 (0%)	1000 (0%)	8000 (5%)	2000 (0%)
	secondary	80	600 (0%)	600 (0%)	600 (0%)	300 (0%)	9600 (5%)	1200 (0%)
	tertiary	50	100 (0%)	100 (0%)	100 (0%)	50 (0%)	2400 (0%)	600 (0%)
	residential	30	50 (0%)	50 (0%)	50 (0%)	25 (0%)	100 (0%)	400 (0%)
	service	30	25 (0%)	25 (0%)	25 (0%)	12 (0%)	50 (0%)	50 (0%)
Night	motorway	130	20,400 (32%)	10,200 (32%)	5100 (32%)	20,400 (32%)	20,400 (37%)	20,400 (32%)
	trunk	110	800 (32%)	400 (32%)	200 (32%)	800 (32%)	6400 (37%)	1600 (32%)
	primary	80	640 (0%)	320 (0%)	160 (0%)	640 (0%)	5120 (5%)	1280 (0%)
	secondary	80	360 (0%)	180 (0%)	180 (0%)	160 (0%)	5760 (0.5%)	720 (0%)
	tertiary	50	50 (0%)	50 (0%)	50 (0%)	25 (0%)	1200 (0%)	300 (0%)
	residential	30	25 (0%)	25 (0%)	25 (0%)	12 (0%)	50 (0%)	50 (0%)
	service	30	12 (0%)	12 (0%)	12 (0%)	6 (0%)	25 (0%)	100 (0%)
**VI (SHV)** **Scenario 7**	**VI (SHV)** **Scenario 8**	**VI (SHV)** **Scenario 9**	**VI (SHV)** **Scenario 10**	**VI (SHV)** **Scenario 11**	**VI (SHV)** **Scenario 12**	**VI (SHV)** **Scenario 13**	**VI (SHV)** **Scenario 14**	**VI (SHV)** **Scenario 15**
33,300 (12.9%)	28,404 (16.2%)	34,315 (11.8%)	35,481 (7.7%)	20,400 (15%)	20,400 (20%)	20,400 (15%)	20,400 (15%)	20,400 (15%)
26,928 (5.4%)	21,012 (7.3%)	26,794 (4.4%)	27,705 (2.3%)	8400 (15%)	8400 (20%)	16,800 (15%)	8400 (15%)	33,600 (15%)
26,928 (5.4%)	21,012 (7.3%)	26,794 (4.4%)	27,705 (2.3%)	4800 (0%)	4800 (5%)	9600 (0%)	4800 (0%)	19,200 (0%)
18,192 (10.7%)	14,652 (13.6%)	18,061 (9.8%)	19,562 (5.7%)	6600 (0%)	6600 (5%)	13,200 (0%)	13,200 (0%)	26,400 (0%)
8928 (6.2%)	7476 (7.6%)	9050 (5.1%)	9717 (2.6%)	2100 (0%)	2100 (5%)	2100 (0%)	4200 (0%)	8400 (0%)
3216 (3.5%)	2400 (4.4%)	3062 (3.1%)	3404 (1.6%)	350 (0%)	350 (5%)	350 (0%)	700 (0%)	1400 (0%)
1098 (2.7%)	768 (3.6%)	1059 (2.4%)	1110 (1.4%)	175 (0%)	175 (5%)	175 (0%)	350 (0%)	700 (0%)
20,400 (11%)	20,400 (11%)	20,400 (11%)	20,400 (11%)	20,400 (11%)	20,400 (16%)	20,400 (11%)	20,400 (11%)	20,400 (11%)
3200 (11%)	3200 (11%)	3200 (11%)	3200 (11%)	1600 (11%)	1600 (16%)	3200 (11%)	1600 (11%)	12,800 (11%)
2000 (0%)	2000 (0%)	2000 (0%)	2000 (0%)	1000 (0%)	1000 (5%)	2000 (0%)	1000 (0%)	8000 (0%)
1200 (0%)	2400 (0%)	2400 (0%)	2400 (0%)	1200 (0%)	1200 (5%)	2400 (0%)	4800 (0%)	9600 (0%)
600 (0%)	600 (0%)	600 (0%)	600 (0%)	600 (0%)	600 (5%)	600 (0%)	1200 (0%)	2400 (0%)
400 (0%)	100 (0%)	100 (0%)	100 (0%)	100 (0%)	100 (5%)	100 (0%)	200 (0%)	400 (0%)
50 (0%)	50 (0%)	50 (0%)	50 (0%)	50 (0%)	50 (5%)	50 (0%)	100 (0%)	200 (0%)
20,400 (32%)	20,400 (32%)	20,400 (32%)	20,400 (32%)	20,400 (32%)	20,400 (37%)	20,400 (32%)	20,400 (32%)	20,400 (32%)
1600 (32%)	1600 (32%)	1600 (32%)	1600 (32%)	800 (32%)	800 (37%)	1600 (32%)	800 (32%)	6400 (32%)
1280 (0%)	1280 (0%)	1280 (0%)	1280 (0%)	640 (0%)	640 (5%)	1280 (0%)	640 (0%)	5120 (0%)
1440 (0%)	1440 (0%)	1440 (0%)	1440 (0%)	720 (0%)	720 (5%)	1440 (0%)	2880 (0%)	5760 (0%)
300 (0%)	300 (0%)	300 (0%)	300 (0%)	300 (0%)	300 (5%)	300 (0%)	600 (0%)	1200 (0%)
50 (0%)	50 (0%)	50 (0%)	50 (0%)	50 (0%)	50 (5%)	50 (0%)	100 (0%)	200 (0%)
25 (0%)	25 (0%)	25 (0%)	25 (0%)	25 (0%)	25 (5%)	25 (0%)	50 (0%)	100 (0%)
